# Urbanization, Trace Metal Pollution, and Malaria Prevalence in the House Sparrow

**DOI:** 10.1371/journal.pone.0053866

**Published:** 2013-01-16

**Authors:** Coraline Bichet, Renaud Scheifler, Michaël Cœurdassier, Romain Julliard, Gabriele Sorci, Claire Loiseau

**Affiliations:** 1 Biogéosciences, CNRS UMR 6282, Université de Bourgogne, Dijon, France; 2 Chrono-Environnement, CNRS UMR 6249, Université de Franche-Comté, Place Leclerc, Besançon, France; 3 Conservation des Espèces, Restauration et Suivi des Populations, UMR 7204 MNHN-CNRS- UPMC, Paris, France; 4 Department of Biology, San Francisco State University, San Francisco, California, United States of America; Monash University, Australia

## Abstract

Anthropogenic pollution poses a threat for the environment and wildlife. Trace metals (TMs) are known to have negative effects on haematological status, oxidative balance, and reproductive success in birds. These pollutants particularly increase in concentration in industrialized, urbanized and intensive agricultural areas. Pollutants can also interfere with the normal functioning of the immune system and, as such, alter the dynamics of host-parasite interactions. Nevertheless, the impact of pollution on infectious diseases has been largely neglected in natural populations of vertebrates. Here, we used a large spatial scale monitoring of 16 house sparrow (*Passer domesticus*) populations to identify environmental variables likely to explain variation in TMs (lead, cadmium, zinc) concentrations in the feathers. In five of these populations, we also studied the potential link between TMs, prevalence of infection with one species of avian malaria, *Plasmodium relictum*, and body condition. Our results show that lead concentration is associated with heavily urbanized habitats and that areas with large woodland coverage have higher cadmium and zinc feather concentrations. Our results suggest that lead concentration in the feathers positively correlates with *P. relictum* prevalence, and that a complex relationship links TM concentrations, infection status, and body condition. This is one of the first studies showing that environmental pollutants are associated with prevalence of an infectious disease in wildlife. The mechanisms underlying this effect are still unknown even though it is tempting to suggest that lead could interfere with the normal functioning of the immune system, as shown in other species. We suggest that more effort should be devoted to elucidate the link between pollution and the dynamics of infectious diseases.

## Introduction

Urbanization and intensive agriculture can drastically affect wildlife populations. Anthropogenic activities produce pollutants known to negatively affect population viability [1]. For instance, organochlorines (OCl) and trace metals (TMs) are known to severely affect breeding success, behavior, and development in birds [2]–[4]. Trace metals particularly increase in concentration in industrialized, urbanized and intensive agricultural areas [5]–[7] and contaminate organisms through air, water and food [8]–[10].

Several biomonitoring programs focused on TMs in birds and top predators since these pollutants may accumulate in organisms [11] and transfer through food chain [12]–[14]. Tissues, including feathers, are often sampled as proxies for total body TM concentrations (avoiding the need to use whole birds for analysis), and the TM levels in such tissues is assumed to be indicative of past TM exposure of the individual [12], [15], [16]. For instance, Scheifler et al. compared urban and rural populations of blackbirds (*Turdus merula*), and they found that lead (Pb) concentration in feathers of birds in urban polluted areas and in their major food item (the community of anecic, epigeous and endogeous earthworms) was greater than in rural areas [7]. TMs are also known to affect physiological parameters such as haematological and oxidative status [17]–[20] potentially resulting in reduced reproductive success [2]. To date, effects of TMs on body condition remain to be fully understood and, in some species, depend on various factors such as sex and populations [21].

TMs are also known to have immunotoxic effects. Although studies on possible effects of pollutants on immune system function remain limited in birds [22], [23], toxicants are know to interfere with immune receptor binding and trigger inappropriate and inhibited immune responses in model species [24]–[28].

In this study, we surveyed sixteen house sparrow (*Passer domesticus*) populations, inhabiting areas with different degrees of urbanization and we determined TM concentrations in feathers. The house sparrow is a widely distributed and sedentary species associated with human settlement, and therefore, is an interesting species in which we can investigate effects of anthropogenic pollution on wildlife. We measured the concentrations of three elements: i) zinc (Zn), which is an essential element, but regulated by organisms because high levels can be toxic, ii) lead (Pb) and cadmium (Cd), two non-essential elements, which have no biological functions identified in birds and can accumulate in organisms.

For a subset of populations (*n* = 5), we also examined the relationship between TM concentrations and the prevalence and intensity of *Plasmodium* infection. *Plasmodium* parasites are the agent of a widespread vector-borne disease of wild birds [29]. House sparrows are infected with several lineages of haemosporidian parasites, including *Plasmodium relictum*. Upon infection, parasitemia usually increases to a peak between one and two weeks post-infection. This acute phase is followed by a chronic infection where parasitemia persists at low levels [29]. Avian malaria has been shown to be harmful to naïve populations and domestic species [30]–[34]. In addition, recent studies based on experimental infections and treatment with antimalarial drugs have also shown that avian malaria parasites can be costly and reduce host fitness in several passerine species [35]–[38].

## Materials and Methods

### Ethics Statement

This fieldwork study involved the sampling of feathers and a small amount of blood of free-ranging birds. The work has been conducted according to relevant national guidelines. The permit to sample birds was delivered by the French Ministère de l’Ecologie et du Développement Durable and the permit to band birds by the Centre de Recherche sur la Biologie des Populations d’Oiseaux at the National Museum of Natural History, Paris.

### Study Sites and Sampling

**Figure 1:**
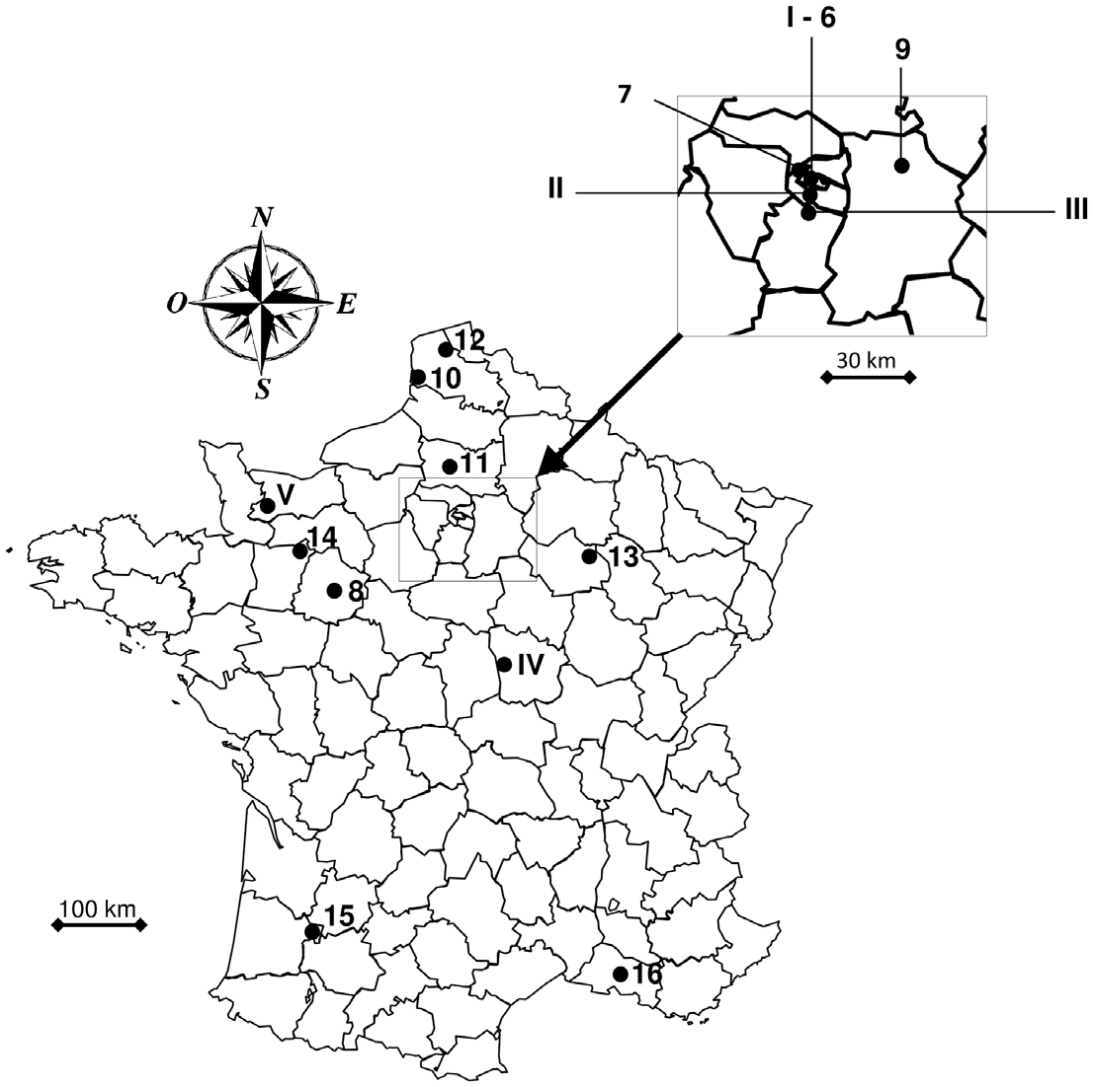
Geographical localization of the 16 house sparrow populations sampled in this study. I - Paris (Jardin des Plantes), II - Cachan, III - Wissous, IV - Cosne-Cours sur Loire, V - Rully, 6 - Paris (Cité internationale universitaire), 7 - Gennevilliers, 8 - Le Mans, 9 - Crégy les Meaux, 10 - Berck, 11 - Thieux, 12 - Seninghem, 13 - Ceffonds, 14 - Crennes, 15 - Réaup-Lisse and 16 - Arles. The five populations in roman numbers were sampled for parasite prevalence and intensity. The zoomed region, in the upper right corner, corresponds to the region Ile de France. Scale bar, 100 km for France and 30 km for the region Ile de France.

We studied 16 populations of house sparrow in France along a gradient of urbanization (Fig. 1, Table 1). Habitat characteristics were obtained from the CORINE (Coordination of information on the environment) Land Cover database using the geographical information system package ArcView 3.2 (ESRI 2000). This is a European geo-referenced land-cover database, based on satellite digital images, that allows classifying landscape units, according to a list of environmental classes [39]. It provides consistent localized geographical information on the land cover of 12 Member States of the European Union. We used the data for year 2000 (http://www.eea.europa.eu/themes/landuse/interactive/clc-download). All the details about the program and the complete methodology can be found in the following internet site: http://www.eea.europa.eu/publications/COR0-landcover. To characterize the surface covered by the different habitat types, we used a circle with a radius of 10 km, centred at the site of capture. We extracted six variables that describe the major habitat characteristics: 1) urban areas, 2) surface covered by intensive agriculture, 3) surface covered by extensive agriculture, 4) woodland, 5) meadow, and 6) shrubland vegetation. These surfaces were expressed in km^2^ and log-transformed for statistical analyses (Table 1).

**Table 1:** Environmental characteristics and TM concentrations for 16 house sparrow populations in France.

		Environmental characteristics (km^2^)						Mean TM concentrations (µg g^−−1^ dry mass) in house sparrow feathers ± standard error					
N°	Population	Urbanarea	Intensive agriculture	Extensive agriculture	Woodland	Meadow	Shrubland	N	Cd	N	Pb	N	Zn
I	Paris, Jardin des Plantes	293.63	0.37	0.61	10.27	0.11	1.32	15	1.51±0.98	15	19.54±2.06	15	188.34±36.56
II	Cachan	265.03	7.73	1.13	21.05	0.44	11.67	13	0.33±0.05	13	8.58±1.18	13	175.84±20.13
III	Wissous	241.66	22.87	1.57	24.57	1.43	15.54	14	0.47±0.15	14	27.06±9.01	14	627.49±88.66
IV	Cosne-Cours sur Loire	17.33	149.76	49.18	48.76	40.98	2.00	18	0.17±0.04	15	3.71±0.86	18	201.65±20.72
V	Rully	4.37	110.92	25.36	4.09	168.94	0.48	12	0.04±0.01	18	3.04±0.20	18	135.72±6.65
6	Paris, Cité internationale universitaire	293.63	0.37	0.61	10.27	0.11	1.32	10	0.20±0.01	10	15.89±2.03	10	167.81±10.18
7	Gennevilliers	289.61	0.88	9.72	4.77	1.02	0.40	18	0.20±0.04	18	18.70±1.67	18	178.33±15.10
8	Le Mans	70.88	67.43	25.68	53.21	93.18	0.35	10	0.65±0.41	10	1.87±0.61	10	161.15±34.64
9	Crégy les Meaux	46.64	192.21	0.87	43.90	7.47	14.54	3	0.63±0.22	3	10.61±4.35	3	503.52±103.08
10	Berck	27.19	57.49	5.49	17.81	32.70	14.34	10	0.26±0.05	10	6.71±2.55	10	188.61±23.99
11	Thieux	16.29	261.85	15.00	20.50	0.51	0	18	0.22±0.03	17	2.79±0.56	18	151.96±11.57
12	Seninghem	10.05	176.75	3.60	34.91	88.35	0.51	18	0.11±0.01	18	2.71±0.32	18	163.20±20.33
13	Ceffonds	6.28	127.87	8.71	101.79	64.29	4.08	18	0.68±0.11	18	3.77±0.71	18	190.47±18.60
14	Crennes	4.14	115.26	5.13	36.02	151.60	2.01	10	0.69±0.10	10	3.99±0.64	10	252.60±68.47
15	Réaup-Lisse	1.62	43.50	55.04	190.09	11.69	11.96	10	0.36±0.12	10	21.08±3.45	10	215.26±22.19
16	Arles	1.42	31.33	55.92	6.91	0	67.99	12	0.07±0.01	18	3.84±0.27	18	106.92±5.14

Adult house sparrows were caught in 2004 and 2005 using mist-nets. The sample size for each population is summarized in Table 1. Each bird was ringed with a numbered metal ring and sexed visually. Two rectrices were collected in all populations for TM measurements. All individuals were, at least, one year old. In five out of the 16 populations (Fig. 1), we measured wing length (±1 mm) and body mass (±0.5 g), and collected a small volume of blood (ca. 20 µl) by brachial vein puncture. Blood was subsequently stored in 500 µl of Queen’s Lysis Buffer (QLB) [40]. Blood samples were not collected for all individuals captured which is the reason for the difference in sample size between the models exploring the environmental predictors of feather TMs and the association between feather TMs and malaria prevalence and parasitemia.

### Parasite Screening

DNA was extracted from blood samples using standard phenol/chloroform protocol (modified from Hillis et al. [41]). In order to detect the presence of malaria parasites, we used a nested polymerase chain reaction (PCR) [42] to amplify a 500 bp fragment of the parasite mitochondrial cytochrome *b* gene. This PCR detects parasites from *Haemoproteus* and *Plasmodium* genera. We sequenced the positive PCR products and identified lineages using the NCBI nucleotide Blast search. For this study, we focused on *Plasmodium relictum* (SGS1 and GRW11 lineages), the predominant parasite in all populations [43]. In addition, for each positive PCR product, we also performed a quantitative PCR to obtain parasitemia (relative quantification) following the protocol described in Cellier-Holzem et al. 2010 [34].

Among the 16 populations, we screened for malaria parasites in five populations along a gradient of urbanization. Paris, Cachan, and Wissous were the most urbanized sites. In Paris, individuals were captured in the botanical garden (*Jardin des Plantes*, hereafter Paris-Jdp). Cachan is located in the suburban area of Paris and individuals from Wissous were caught in an industrialized area near the Orly International Airport. In contrast to these highly urbanized sites, the other populations (Cosne-Cours sur Loire [hereafter Cosnes-C/L] and Rully) can be considered as rural, with less than 6% of urbanization (Table 1). All populations were sampled in spring/summer except Cosne-C/L, which was partly sampled in fall/winter.

### TMs Analysis

In order to remove exogenous contamination, feathers were washed (1 min in acetone) and then rinsed (1 min with deionised water, 18.2 MΩ cm^−2^) three times in an ultrasonic bath. Before analysis, washed feathers were dried in an oven at 60°C until constant dry mass was achieved. Dry mass was determined to the nearest 0.0001 g using an electronic balance (Mettler Toledo AB 54). Samples were digested in a mixture of 2 ml HNO_3_ (68%) and 2 ml H_2_O_2_ (30%) for 48 h in an oven at 60°C. Samples were diluted by adding 11 ml deionised water and were stored at −20°C until analysis. All reagents were of analytical grade and obtained from Carlo Erba (Val de Rueil, France).

Total Cd and Pb concentrations were measured by furnace atomic absorption spectrophotometry (AAS, Varian 220Z, Les Ulis, France), Zn by flame AAS (Varian 220FS, Les Ulis, France). All concentrations are expressed in micrograms per gram on a dry mass basis (µg g^−1^ dm).

Validity of analytical methods was checked by means of standard biological reference material (TORT-2, lobster hepatopancreas, and DOLT-3, dogfish liver, from the National Research Council of Canada–Institute for National Measurement Standard, Ottawa, ON, Canada). Recoveries for Cd, Pb, and Zn concentrations from the TORT-2 and DOLT-3 reference materials were 126±24 and 103±12%, 169±32 and 175±48%, and 98±5 and 100±8%, respectively. Detection limits (DL) calculated using blank values and average dry mass of feathers were 0.05 and 1.59 µg g^−1^ for Cd and Pb, respectively. DL could not be calculated for Zn because blank measurements gave systematically negative values. For statistical analyses, values under DL were replaced by half of the DL [44].

One individual gave values below the DL for Cd and Pb and was excluded from the analyses.

### Statistical Analyses

First, we tested the correlations between TM concentrations and environmental variables using Pearson’s correlations. Since some of these habitat variables were correlated among them (Table 2), and to avoid co-linearity in the statistical models, we summarized the information carried by these descriptors across major axes, using a principal component analysis (PCA). Axes 1 to 3 explained 49%, 20%, and 16% of the variance, respectively, and were used in the following statistical analyses. Axis 1 represented a major gradient of urbanization with a positive load for intensive (0.51), extensive agriculture (0.40), woodland (0.34), and meadows (0.45), and a negative load for urban areas (−0.51). Axis 2 strongly loaded for shrubland vegetation (0.87), whereas axis 3 loaded for woodland (0.70), and negatively for extensive agriculture (−0.61). The predictive power of axes 1 to 3 to explain the amount of TMs incorporated into sparrow feathers was estimated using linear mixed models (LMM) with a normal distribution of errors. To this purpose, TM concentrations were log-transformed and the site was included in the models as a random variable.

**Table 2:** Pearson’s correlation coefficient matrix among the environmental variables used to characterize 16 house sparrow populations in France. * and ** indicates P values ≤0.05 and ≤0.01, respectively.

	Urban areas	Intensive agriculture	Woodland	Extensive agriculture	Meadow
Urban areas					
Intensive agriculture	−0.7016**				
Woodland	−0.3292	0.4896*			
Extensive agriculture	−0.6895**	0.4231	0.1710		
Meadow	−0.5285*	0.6531**	0.3919	0.3224	
Shrubland	−0.1856	0.0211	0.1310	0.0175	−0.2897

For a subset of five populations, we also assessed the association between prevalence (defined as a binary variable, 0 for non-infected and 1 for infected birds) or parasitemia (using infected individuals only) of malaria parasites, and sex, site, and TMs, using a generalized linear model (distribution of errors: binomial, link: logit) and a general linear model (distribution of errors: Gaussian, link: identity), respectively. To perform these analyses, several models were constructed: the null model, five models each containing one of the five variables (sex, site, Cd, Pb, or Zn), a model containing all the variables, a model containing only sex and site, a model containing all TMs (Cd, Pb, Zn), and a model containing non-essential TMs (Cd, Pb).

Body condition was assessed as the residuals of a linear regression of body mass on wing length (*F*_1,64_ = 9.34, *P* = 0.003). Body condition was then modelled (linear model) using sex, site, infection status (as a binary variable), or TMs, as explanatory variables. First order interactions between the infection status and other variables were also added in the models. More complex interactions could not be tested due to the limited sample size. The various models were built as described above (null model, models with each one of the six variables, models with sex and site, models with all or non-essential TMs). In the models exploring the associations between TMs and malaria, site (n = 5) was included as a fixed factor because the comparisons of models with site as a fixed factor and those with site as a random factor showed that the former were more parsimonious.

We used the package lme4 [45], implemented in R 2.15.0 to run all LMMs. We used the information-theoretic (IT) approach as it has recently been suggested as being more appropriate for observational studies [46]. Model support was assessed using the corrected version of Akaike Information Criterion (AICc) for small sample sizes, and ΔAIC was used to infer support for models in the candidate set [47]. We calculated the Akaike weights (*ω*) for each model, which is the probability that a model is selected as the best model in a model set [46]. We deemed that there was essentially no evidence in support to a model when its ΔAIC value was greater than 10 [46]. It is also worth noting that when a fitted parameter was added to a model, a penalty of 2 is added to the model’s AIC value [46]. Thus, we considered that a variable improved the fit of the model only when the ΔAIC was lower than 2. The maximized log-likelihood (LL) and number of estimated parameters (K) were also calculated and reported in the text.

Selected linear models were checked graphically for homogeneity of variance, normality of error and linearity/additivity. The leverage was evaluated by looking at plots of the standardized residuals versus leverage. Model outputs were satisfactory, and transformations of the measured variables did not bring significant improvement. Non-transformed variables were therefore used in the statistical analyses.

Model coefficients and confidence intervals are given for the linear predictor for linear models and for the exponential of the linear predictor in linear/generalized models.

## Results

### Environmental Variables Associated with TM Concentrations

Average TM concentrations varied between 0.04 and 1.51 µg g^−1^ for Cd, 1.87 and 27.06 for Pb, and 106.92 and 627.49 for Zn (Table 1).

**Figure 2:**
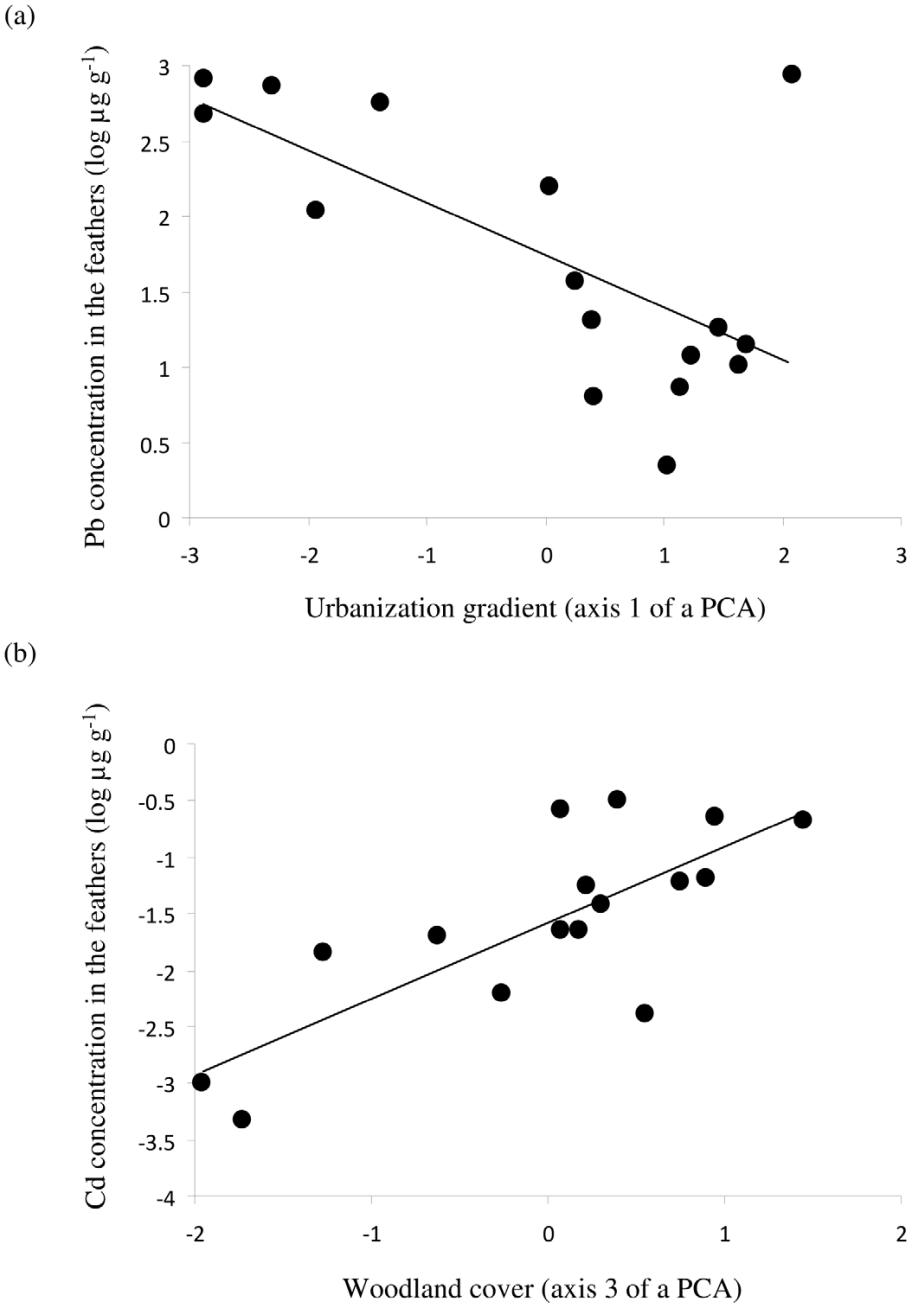
(a) Negative correlation between Pb concentration in feathers and the axis 1 of the principal component analysis on environmental variables (see materials and methods for more details). (b) Positive correlation between Cd concentration in feathers and the axis 3 of the principal component analysis. Each point represents a house sparrow population.

Axis 1 of the PCA describes a gradient of urbanization/natural landscapes, with positive values indicating prevailing natural habitats, negative values urbanized areas. According to Akaike parameters, Pb concentrations were best explained by a model including the axis 1 and the axis 2 (LL: 4.94, K: 3, AICc: −12.21, ΔAICc: 0.00, ω: 0.35). A competitive model (ΔAICc: 0.28) included only the axis 1. Pb concentrations were negatively associated with this axis, showing that high Pb concentrations were tightly associated with heavily urbanized habitats (Fig. 2a).

Cd concentrations were best explained by the model including only the axis 3 (LL: 152, K: 2, AICc: −308.88, ΔAICc: 0.00, ω: 0.51). The result was similar for Zn, where the best model also included the axis 3 (LL: 43.43, K: 2, AICc: −88.34, ΔAICc: 0.00, ω: 0.46). Feather concentrations of Cd and Zn were positively associated with axis 3 of the PCA. Axis 3 describes woodland areas, indicating that sparrows sampled in areas with a large woodland coverage tended to have higher concentrations of Cd and Zn in their feathers (Fig. 2b).

### TMs and Risk of Malaria Infection

**Figure 3:**
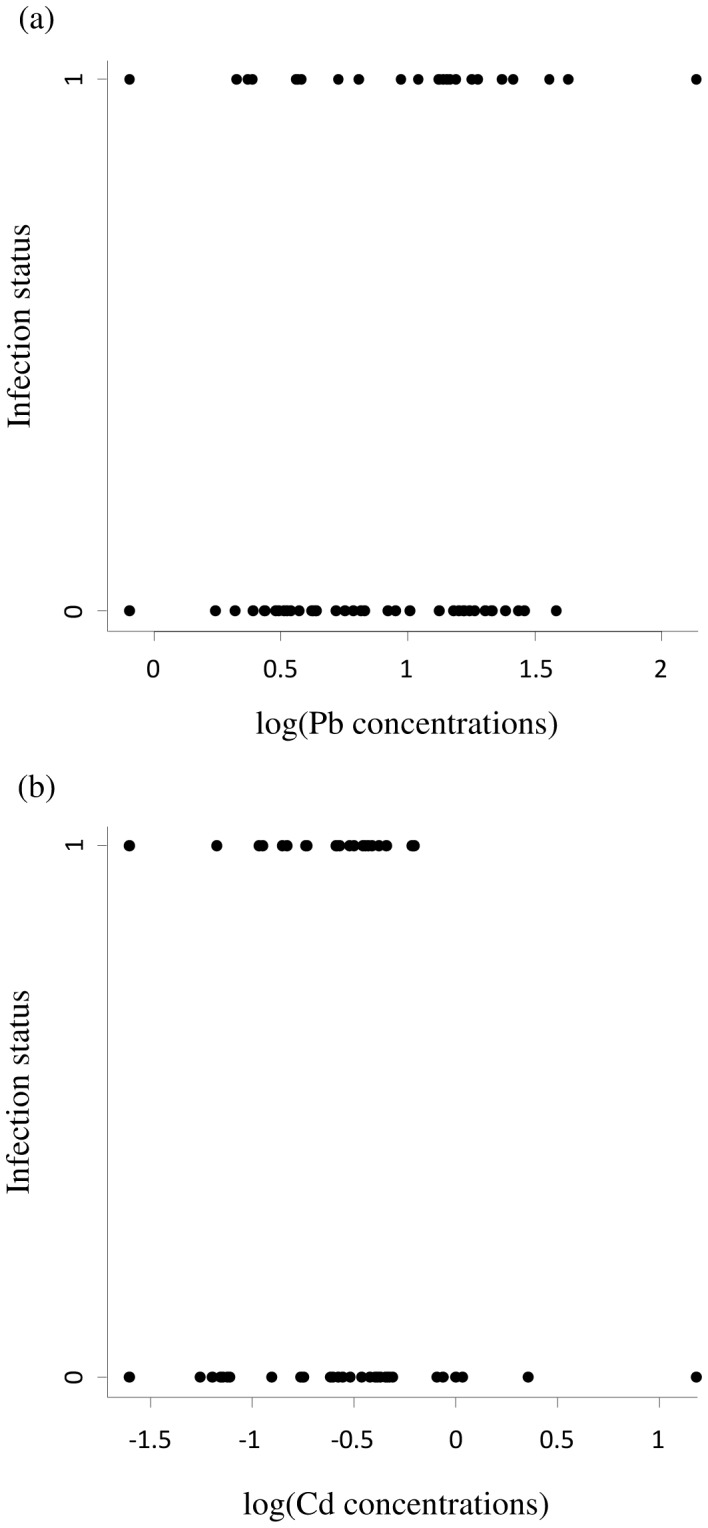
Association between infection with *Plasmodium* (0 = non-infected, 1 = infected) and Pb and Cd concentration in feathers of house sparrows. Metal concentrations are logged to facilitate the reading of the figure.

*Plasmodium* prevalence was 25% in Rully, 36% in Paris-JdP, 43% in Wissous, 44% in Cosne-C/L and 46% in Cachan. According to Akaike parameters, *Plasmodium* prevalence was best explained by a model including the two non-essential metals, Cd and Pb (LL: −39.83, K: 3, AICc: 86.05, ΔAICc: 0.00, wic: 0.44). According to the exponential of the linear predictor of the variables and their confidence intervals, prevalence is negatively associated to Cd concentrations (exponential value of the linear predictor: 0.14, confidence interval: 0.01/0.95) and positively associated with Pb concentrations (1.08, 1.01/1.18) (Fig. 3). In addition to parasite prevalence, we assessed parasitemia of infected birds. Parasitemia was very low as expected for chronic malaria infections. The null model was found to be the best (LL: 140.48, K: 2, AICc: −276.42, ΔAICc: 0.00, wic: 0.31), most likely because of limited statistical power and/or low variability in parasitemia.

### Body Condition

According to Akaike criterion, body condition was best fitted by a model including site, the infection status, and their interaction (LL: −118.52, K: 11, AICc: 263.93, ΔAICc: 0.00, wic: 0.44). A competitive model (ΔAICc: 0.80) included Zn concentration, the infection status and their interaction. The linear predictor of the variables and their confidence intervals showed that i) individuals in Wissous and Cosne-C/L were in better body condition compared to Paris, ii) infected birds were in worse condition compared to non-infected individuals in Wissous, iii) the relationship between body condition and Zn concentration was positive in non-infected birds and negative in *Plasmodium* infected birds.

## Discussion

It has been established that pollution and especially contamination by TMs can affect human and wildlife health. However, there is little information about the effect of pollutants on the prevalence of infectious diseases. With the current increase in urbanization, it seems relevant to investigate how pollution can be linked with the susceptibility of free-living animals to pathogens. Here, we studied the association between urbanization and TM concentrations in sixteen populations of the house sparrow. In addition, we explored the correlation between TMs and malaria prevalence. We found that i) sparrows living in highly urbanized areas had a higher Pb concentration in their feathers, ii) Cd and Zn concentrations were associated with sites mostly covered by woodlands, iii) Pb concentrations might be associated with higher *Plasmodium* prevalence, while Cd concentrations tended to be negatively correlated with prevalence, iv) body condition was similar or higher in infected than in non-infected birds in all the populations except at Wissous, where sparrows have high Zn concentration in their feathers.

Our finding that Pb concentration follows an urbanization/natural habitat gradient corroborates previous results gathered on a variety of species, including the house sparrow [5]–[7], [16], [48], and further shows that, even though Pb is no longer used as a gasoline additive, it persists in urban environments. All urban populations had high values of Pb in the feathers. There is, however, one notable exception to this pattern. A rural population (Réaup-Lisse) had among the highest values of Pb. Although we do not know the local source of this pollution, this shows that other factors in addition to urbanization may determine Pb contamination in birds. Pb feather contamination is generally thought to occur through the incorporation of the TMs ingested with food [49] during feather growth [12]. TM concentrations have also been reported to increase with bird age [50], [51], suggesting a time-dependent accumulation. However, samples analyzed here only concerned individuals more than 1-year old, which should reduce the variance due to differential time-dependent exposure.

Few studies have assessed *P. domesticus* or other sparrow species exposure to TMs using feathers and, to our knowledge, investigations on rural populations living in uncontaminated areas have never been reported. Thus, no reference values have been proposed for TM concentrations in sparrow feathers in rural habitats. Data from other species should be generalized to sparrows with care, because of interspecies variations in TM accumulation. Burger [12] determined a median Cd level in feathers of 0.1 µg/g from avian studies worldwide, and 4.5 µg/g of Pb were associated with behavioral abnormalities in birds that can cause lowered survival [52], [53]. In our study, 87% of the sparrows had higher Cd residues in feathers than the median values reported above, and 50% were over the Pb concentrations known to have detrimental effects. Considering only those studies performed on sparrows, feather residues in urban populations of *P. domesticus* in Palestine were 0.02±0.00 µg/g for Cd, 8.1±1.3 for Pb, and 54.9±5.3 µg/g for Zn [48]. All the sparrow populations we studied had higher Cd (from 2 to 75-fold) and Zn (from 1.9 to 11.5-fold) concentrations, while 44% of them exhibited higher Pb residues, mainly among those living in the most urbanized environments. Pan et al. (2008) reported TM concentrations in ventral feathers of the tree sparrow (*Passer montanus*) in Beijing [54]. In their sampling site where the traffic was an important source of pollutants, concentrations in male adult tree sparrows reached 0.74±0.41 µg/g and 13.63±2.33 µg/g of Cd and Pb, respectively. These values are in the same range, even if slightly higher, than those we measured in urban house sparrows. The sparrow populations studied here exhibited a wide range of exposure to TMs. Most of the populations living in the most urbanized areas exhibited high exposure level. The population living in the *Jardin des Plantes* (Paris-Jdp), the most urbanized site, is exposed to high levels of both Cd and Pb. Other populations, such as those of Wissous, Gennevilliers, and Réaup-Lisse, suffered high Pb exposure, despite different degrees of urbanization. Finally, populations inhabiting in rural areas (*e.g.* Berck, Thieux, and Arles) generally exhibited low to moderate concentrations of TMs.

The association between Cd and Zn concentrations with sites mainly covered by woodlands is more surprising. Because Cd and Zn median concentrations are of the same order of magnitude for most of the 16 sites and are below 1 and 400 µg g^−1^, respectively, it is possible that they represent mainly a transfer from geochemical background to sparrow feathers through food. Accordingly, the observed association between Cd and Zn concentrations and woodlands might be due to a higher bioavailability of these two TMs in areas with low pH (which is usually the case in woodlands compared to other habitat types) [55].

Besides identifying the environmental determinants of TM pollution at a large spatial scale, the aim of our study was to investigate the potential link between TM concentrations and malaria prevalence. In agreement with the hypothesis that TMs can disrupt the normal functioning of the immune response and enhance risk of contracting infectious diseases, our results suggest that malaria prevalence could be positively correlated with Pb concentrations. We should however note that, although the overall sample size was quite large, the number of birds sampled for malaria parasites per population ranged from 9 to 16, which might have resulted in an underestimate of the prevalence of the infection.

Pb concentrations were not correlated with parasitemia (nor were the two other TMs). Even though this result might suggest that parasitemia is not affected by environmental pollution, the reduced statistical power and/or the low among-individual variability in parasitemia prevent us to draw a firm conclusion. It should also be noted that birds suffering from high parasitemia might be more difficult to capture because of reduced mobility, possibly biasing the sample towards birds with chronic parasitemia [29].

Given the increasingly rate at which human activity impacts on natural habitats and the well-known effect of pathogens on natural populations of hosts [56], it is surprising to note that the study of the consequences of environmental pollution for the dynamics of infectious diseases has been largely neglected. Among the rare studies where pollutants and infectious diseases have been monitored, Mashima et al. reported that the liver concentration of Cd was higher in avian cholera infected oldsquaws (*Clangula hyemalis*), compared to apparently healthy individuals [57]. However, it should be noted that in this study, avian cholera infected and healthy individuals were collected at different time points (1994 and 1985–87, respectively), which makes difficult to draw a firm conclusion on the link between Cd and susceptibility to avian cholera.

The effects of TMs, notably Cd, Pb and mercury (Hg), on the immune system of birds have been widely demonstrated both *in vitro* and *in vivo*, although the mechanisms involved remain under debate. Metals are generally reported to depress the immune response [23], [58]–[61], resulting in increased susceptibility to infectious diseases and parasites [62]. Other pollutants have also been reported to have similar immunotoxic effects. For example, Camplani et al. (1999) studied the impact of radioactive pollution on barn swallows (*Hirundo rustica*) living in the irradiated site of Chernobyl. They found that lymphocyte and immunoglobulin concentrations were depressed and spleen size was reduced [63].

It should also been noted that subchronic exposure to Pb or Zn may enhance immune functions [64], [65], Zn being even considered essential in all aspects of immunity [66], [67]. This might explain our finding of a negative correlation between malaria prevalence and Cd concentrations. Alternatively, a negative correlation between pollutants and prevalence might arise if the pollutant exerts its toxic effect on both the host and the parasite. In addition to this, the timing of exposure to pollutants and the infectious agents might affect the sign of the association [62]. Experimental work on lab mice showed that exposure to lead or nickel enhanced mice resistance to a subsequent infection with *Klebsiella pneumoniae*. However, when the pollutant exposure intervened after the infectious challenge, mouse resistance was impaired [68].

To date, no biological function has been identified in birds for both Cd and Pb. The best evidence in support to the idea that TMs can depress the immune response in natural populations of birds comes from Snoeijs et al. [22]. They measured the humoral immune responsiveness of great tits (*Parus major*) (i.e., the proportion of birds that produced detectable antibodies following injection of sheep red blood cells) along a pollution gradient near a metallurgic smelter, and found that birds sampled from the site farthest away from the smelter complex had a significantly higher immune responsiveness than birds from the areas closest to the smelter [22].

Contrary to what could have been expected, body condition was similar or even better in infected birds than in non-infected individuals, except in Wissous (the site with the highest Zn concentration). Previous work has mostly failed to find an association between TMs and body condition in wild passerines [21], whereas the association between malaria infection and body condition seems to vary depending on environmental conditions. For instance, physiological condition of malaria infected great tits was poorer than for non-infected individuals, even though year and season of sampling had a strong effect on the strength of the association [69]. Finding a statistical support for the association between malaria infection and body condition usually requires either an experimental approach either field studies with very large sample sizes [70], [71]. As mentioned above, potential bias in the sampling of heavily infected birds [29], [72] further contribute to the difficulty to properly assess the relationship between body condition, TM concentrations, and the infection with *Plasmodium*.

Given the correlative nature of the results reported here, we cannot infer the causality relationship between TM pollution, parasitism and body condition. Although it is tempting to speculate that pollution makes organisms more susceptible to infectious diseases by altering their immune functions, we cannot discard the possibility that sparrows using polluted spots are more exposed to malaria-infected mosquitoes. This would also result in a positive correlation between prevalence of infection and concentration of TMs in the feathers with no role for TM immunotoxicity. Malaria parasites and their vectors can be affected by environmental characteristics, such as temperature, humidity and altitude [73]–[77].

To conclude, the multiple interactions between pollutants, pathogens and environment are complex and still difficult to disentangle. However, in the current context of emerging infectious diseases and rapid urbanization, understanding the consequences of multi-pollutants and multi-pathogens interactions will be an essential step to predict population persistence and wildlife adaptation to human-made habitats.
